# Variation of genes encoding nitric oxide synthases and antioxidant enzymes as potential risks of multiple sclerosis development: a preliminary study

**DOI:** 10.1038/s41598-022-14795-6

**Published:** 2022-06-22

**Authors:** Paulina Wigner, Angela Dziedzic, Ewelina Synowiec, Elzbieta Miller, Michal Bijak, Joanna Saluk-Bijak

**Affiliations:** 1 Department of General Biochemistry, Faculty of Biology and Environmental Protection, University of Lodz, Pomorska 141/143, 90-236 Lodz, Poland; 2 Laboratory of Medical Genetics, Faculty of Biology and Environmental Protection, University of Lodz, 141/143 Pomorska Street, 90-236 Lodz, Poland; 3 Department of Neurological Rehabilitation, Medical University of Lodz, Milionowa 14, 93-113 Lodz, Poland; 4 Biohazard Prevention Centre, Faculty of Biology and Environmental Protection, University of Lodz, Pomorska 141/143, 90-236 Lodz, Poland

**Keywords:** Genetic variation, Risk factors, Multiple sclerosis, Genetics research

## Abstract

Multiple sclerosis (MS) is a neurodegenerative disease characterized by a variable clinical course and diverse pathophysiology, including nitrative and oxidative stresses as well as inflammation. We aimed to detect the potential association between five selected single-nucleotide polymorphisms (SNPs) in genes encoding nitric oxide synthetases as well as antioxidant enzymes and the development of MS in a Polish population. Genomic DNA was isolated from peripheral blood collected from 142 MS patients and 140 controls. Using Taq-Man^®^ probes, we genotyped the following SNPs: rs1879417 in *NOS1*, and rs2297518 in *NOS2* as well as rs4880 in *SOD2*, rs7943316 in *CAT*, rs713041 in *GPX4*. In the case of rs2297518, the C/C genotype and C allele SNP were associated with an enhanced occurrence of MS, while the C/T, T/T genotypes, and T allele of the same polymorphism reduced this risk. Moreover, the C/C homozygote and C allele of the rs4880 SNP reduced MS risk, while the T allele increased the risk. In addition, the A/T heterozygote of rs7943316 polymorphism was associated with an increased risk of MS occurrence. We also detected that the C/C genotype and C allele of rs713041 decreased the risk of MS, whereas the T/T genotype and T allele increased this risk. In conclusion, the results of our study suggest some links between polymorphic variability in the nitrative/oxidative stress-related genes and the risk of MS development in the Polish population.

## Introduction

Multiple sclerosis (MS) is a neurodegenerative autoimmune disease characterized by a variable clinical course and diverse pathophysiology, including inflammation associated with demyelination, neurons damage as well as loss of blood–brain barrier (BBB) integrity^1^. MS is the most common diverse neurological disabling disease among young adults^2^. The incidence of MS is alarmingly growing worldwide, together with the socio-economic effect of the disease. According to current epidemiological data, a total of 2.8 million people suffer from MS globally (mean 35.9 per 100,000 people), a 30% increase over several years^3^. It is known that MS is much more common in women than in men, and its prevalence ratio has dangerously enhanced during the last decade from 2.3:1 to 3.5:1^4,5^.

Epidemiological data indicate that genetic and environmental factors are significant for the etiology of MS, but the fundamental cause of the disease still residues unrevealed^6^. The processes underlying the pathomechanism of MS are mainly abnormalities in the immune response, including the massive influx of immune cells (especially T-cells and B-cells) into the central nervous system (CNS) combined with the dysfunction of myelin-producing cells^7^. However, in addition to inflammation, numerous studies also confirmed the crucial role of nitrative and oxidative stress markers in the development of MS^8–10^. Despite the existence of physiological antioxidant mechanisms, CNS cells, especially neurons, are not fully protected against RNS (reactive nitrogen species) and ROS (reactive oxygen species) overproduction. The brain exhibits high oxygen consumption and robust free radical production due to its intense aerobic metabolism^11^. In the CNS, a chronic inflammatory state and disturbed redox status lead to progressive demyelination and disturbed neuronal transmission, which is the main cause of psycho-motor disability^12^. It is well-documented that MS pathophysiology is strictly associated with excessive RNS and ROS generation, together with the weakened antioxidant defense system, headed with the aberrant level of the free radical scavenging enzymes^13,14^. A high level of RNS and ROS induce mitochondrial damage that initiates focal axon degeneration^15^. In turn, the reduced levels of antioxidants may contribute to an increased lipoxygenase activity, which triggers leukotrienes generation, thereby increasing the immune-inflammatory processes in brain tissue^16^. The most important antioxidant enzymes are nitric oxide synthase 1 (NOS), nitric oxide synthase 2 (NOS2), superoxide dismutase 2 (SOD2), catalase (CAT), and glutathione peroxidase 4 (GPX4). The level of these enzymes may therefore reflect the status of an antioxidant defense line, which may influence the elimination rate of free radicals, such as superoxide (O_2_^−**·**^) or hydroxyl radical (OH·) and non-radical molecules such as hydrogen peroxide (H_2_O_2_)^17^. On the other hand, other studies suggested that excess RNS and ROS may stimulate the heightened activity of T-cell via an arachidonic acid cascade, or produce direct/indirect damage to the BBB or myelin^18^. Therefore, the aforementioned mechanism indicates that intensification of nitrative and oxidative stress, caused, among others, by decreased levels and activity of enzymatic antioxidants and/or excessive pro-oxidative enzyme activity, may play an important role in MS etiology.

Moreover, currently, there is a growing interest in finding the relationship between the genetic base of nitrative as well as oxidative stresses and MS pathogenesis. It has been documented that genetic factors, such as single-nucleotide polymorphisms (SNPs) have fundamental effects on nitrative and oxidative stresses pathways in various neurodegenerative diseases^19–21^. However, despite the abundant evidence that nitrative and oxidative stresses contribute to the pathomechanism of MS, the association of MS occurrence and polymorphisms in genes, encoding NOS, as well as antioxidant enzymes, has not been studied so far.

Therefore, we aimed to detect the potential association between five polymorphisms in genes encoding nitric oxide synthetases as well as antioxidant enzymes and the development of MS in a Polish population.

## Results

### Single nucleotide polymorphisms of nitrative and oxidative stresses-related genes as the risk of MS occurrence

#### The g.117803515 C > T (rs1879417) SNP of the NOS1 and the risk of MS occurrence

Our findings show that the g.117803515 C > T—*NOS1* SNP was not significantly associated with MS (Table 1).

**Table 1:** Distribution of genotypes and alleles of the g.117803515 C > T—*NOS1* (rs1879417), c.1823 C > T (p.Ser608Leu)—*NOS2* (rs2297518), c.47 C > T—*SOD2* (rs4880), c.-89 A > T—*CAT* (rs7943316), c.660 T > C—*GPX4* (rs713041) and ORs with 95% Cis in patients with MS and healthy volunteers.

Genotype/allele	Control (n = 140)		MS (n = 142)		Crude OR (95% CI)*	*p*	Adjusted OR (95% CI)*	*p*
	Number	Frequency	Number	Frequency				
**g.117803515 C > T**—***NOS1* (rs1879417)**								
C/C	23	0.164	35	0.246	1.664 (0.924–2.995)	0.090	1.753 (0.967–3.176)	0.064
C/T	78	0.557	70	0.493	0.773 (0.484–1.235)	0.281	0.760 (0.474–1.218)	0.254
T/T	39	0.279	37	0.261	0.913 (0.539–1.545)	0.733	0.895 (0.527–1.521)	0.683
χ^2^ = 1.505; *p* = 0.220								
C	124	0.443	140	0.493	1.238 (0.879–1.743)	0.221	1.270 (0.899–1.795)	0.175
T	156	0.557	144	0.507	0.808 (0.574–1.137)	0.221	0.787 (0.557–1.112)	0.175
**c.1823C > T (p.Ser608Leu)**—***NOS2* (rs2297518)**								
**C/C**	**60**	**0.429**	**99**	**0.697**	**3.070 (1.880–5.011)**	**< 0.001**^**0.996**^	**3.131 (1.909–5.135)**	**< 0.001**^**0.996**^
*C/T*	*48*	*0.343*	*29*	*0.204*	*0.492* (*0.288–0.841*)	*0.010*^**0.740**^	*0.488* (*0.285–0.839*)	*0.009*^**0.748**^
*T/T*	*32*	*0.229*	*14*	*0.099*	*0.369* (*0.187–0.727*)	*0.004*^**0.836**^	*0.361* (*0.182–0.715*)	*0.003*^**0.849**^
χ^2^ = 20.294; *p* < 0.001								
**C**	**168**	**0.600**	**227**	**0.799**	**2.101 (1.501–2.941)**	** < 0.001**^**0.982**^	**2.131 (1.518–2.992)**	**< 0.001**^**0.985**^
*T*	*112*	*0.400*	*57*	*0.201*	*0.476* (*0.340–0.666*)	< *0.001*^**0.982**^	*0.469* (*0.334–0.659*)	< *0.001*^**0.985**^
**c.47 C > T (p.Val16Ala)**—***SOD2* (rs4880)**								
*C/C*	*38*	*0.271*	*23*	*0.162*	*0.519* (*0.290–0.928*)	*0.027*^*0.600*^	*0.484* (*0.268–0.874*)	*0.016*^**0.678**^
T/C	74	0.529	83	0.585	1.255 (0.784–2.009)	0.345	1.240 (0.772–1.991)	0.374
T/T	28	0.200	36	0.254	1.358 (0.775–2.380)	0.284	1.474 (0.833–2.609)	0.183
χ^2^ = 4.250; *p* = 0.039								
*C*	*150*	*0.536*	*129*	*0.454*	*0.689* (*0.483–0.985*)	*0.041*^**0.595**^	*0.647* (*0.449–0.932*)	*0.019*^**0.729**^
**T**	**130**	**0.464**	**155**	**0.546**	**1.451 (1.015–2.072)**	**0.041**^**0.595**^	**1.546 (1.073–2.227)**	**0.019**^**0.730**^
**c.-89 A > T**—***CAT* (rs7943316)**								
A/A	24	0.171	13	0.092	0.487 (0.237–1.001)	0.050	0.500 (0.242–1.031)	0.060
**A/T**	**97**	**0.693**	**118**	**0.831**	**2.180 (1.236–3.843)**	**0.007**^**0.774**^	**2.200 (1.243–3.894)**	**0.007**^**0.783**^
T/T	19	0.136	11	0.077	0.535 (0.244–1.170)	0.117	0.508 (0.230–1.119)	0.093
χ^2^ = 0.139; p = 0.709								
A	145	0.518	144	0.507	0.913 (0.565–1.475)	0.709	0.942 (0.581–1.528)	0.809
T	135	0.482	140	0.493	1.096 (0.678–1.771)	0.709	1.062 (0.654–1.722)	0.809
**c.660 T > C**—***GPX4* (rs713041)**								
*C/C*	*57*	*0.407*	*39*	*0.275*	*0.551* (*0.335–0.909*)	*0.019*^**0.648**^	*0.548 *(*0.331–0.906*)	*0.019*^**0.656**^
T/C	65	0.464	72	0.507	1.187 (0.744–1.894)	0.473	1.215 (0.759–1.947)	0.417
**T/T**	**18**	**0.129**	**31**	**0.218**	**1.893 (1.003–3.573)**	**0.049**^**0.505**^	**1.838 (0.970–3.481)**	**0.062**^**0.466**^
χ^2^ = 7.223; *p* = 0.007								
*C*	*179*	*0.639*	*150*	*0.528*	*0.628* (*0.445–0.886*)	*0.008*^**0.773**^	*0.632* (*0.447–0.893*)	*0.009*^**0.762**^
**T**	**101**	**0.361**	**134**	**0.472**	**1.592 (1.129–2.246)**	**0.008**^**0.773**^	**1.583 (1.120–2.237)**	**0.009**^**0.762**^

*p-*values were obtained from logistic regression analyses with the codominant, dominant and recessive models without Bonferroni correction*. p* < 0.05 along with corresponding ORs are bold and in bold (for the genotypes/alleles contributing to the MS development) or in italics (for the genotypes/alleles with a protective effect); *OR adjusted for gender. Statistical power (1 − β) for significant comparisons is given in superscripts.

#### The c.1823 C > T (p.Ser608Leu) (rs2297518) SNP of the NOS2 and the risk of MS occurrence

We also detected that the C/C homozygote (Crude OR 3.070; 1.880–5.011 95% CI; *p* < 0.001) and the C allele (Crude OR 2.101; 1.501–2.941 95% CI; *p* < 0.001) of the c.1832C > T—*NOS2* SNP were associated with an elevated risk of MS, while the heterozygote (Crude OR 0.492; 0.288–0.841 95% CI; *p* < 0.05), the T/T homozygote (Crude OR 0.369; 0.187–0.727 95% CI; *p* < 0.001) and the T allele (Crude OR 0.476; 0.340–0.666 95% CI; *p* < 0.001) of the same polymorphism were associated with a decreased risk of this disease (Table 1).

#### The c.47 C > T (rs4880) SNP of the SOD2 and the risk of MS occurrence

As shown in Table 1, the C/C genotype of the c.47 C > T—*SOD2* SNP was linked with a decreased frequency of MS occurrence (Crude OR 0.519; 0.290–0.928; 95% CI; *p* < 0.05). Moreover, the C allele was associated with a reduced MS risk (Crude OR 0.689; 0.483–0.985 95% CI; *p* < 0.05), whereas the T allele was associated with an increased risk of the disease occurance (Crude OR 1.451; 1.015–2.072 95% CI; *p* < 0.05).

#### The c.-89 A > T (rs7943316) SNP of the CAT and the risk of MS occurrence

We found, that the A/T heterozygote was associated with a decreased occurrence of MS (Crude OR 2.180; 1.236–3.843 95% CI; *p* < 0.01) (Table 1).

#### The c.660 T > C (rs713041) SNP of the GPX4 and the risk of MS occurrence

In the case of the c.660 T > C—*GPX4* polymorphism, the C/C homozygote (Crude OR 0.551; 0.335–0.909 95% CI; *p* < 0.05) and the C allele (Crude OR 0.628; 0.445–0.886 95% CI; *p* < 0.01) were associated with a reduced risk of MS development, while genotype T/T (Crude OR 1.893; 1.003–3.573 95% CI; *p* < 0.05) and allele T (Crude OR 1.592; 1.129–2.246 95% CI; *p* < 0.01) of the same SNP were associated with an increased risk of the disease (Table 1).

### Association between combined genotypes of *NOS1*, *NOS2*, *SOD2*, *CAT*, *GPX4* polymorphisms and the risk of MS occurrence—gene–gene interaction

We also studied the correlation between MS occurrence and combined genotypes of the tested polymorphisms localized in genes, encoding enzymes associated with oxidative/nitrative stress, and the results are presented in Table 2.

**Table 2:** Gene–gene interactions of studied oxidative stress-related polymorphisms and MS risk.

Combined genotype	Control (n = 140)		MS (n = 142)		Crude OR (95% CI)*	p*	Adjusted OR (95% CI)**	p**
	Number	Frequency	Number	Frequency				
**c.47 C > T (p.Val16Ala)**—***SOD2* (rs4880) AND c.-89 A > T**—***CAT* (rs7943316)**								
T/T–A/A	4	0.029	4	0.028	0.986 (0.242–4.020)	0.999	1.005 (0.244–0.135)	0.999
T/T–A/T	23	0.164	30	0.211	1.363 (0.746–2.487)	0.529	1.504 (0.814–2.778)	0.349
T/T–T/T	1	0.007	2	0.014	1.986 (0.178–22.151)	0.821	1.790 (0.158–20.259)	0.869
T/C–A/A	15	0.107	9	0.063	0.564 (0.238–1.335)	0.349	0.572 (0.241–1.361)	0.371
T/C–A/T	51	0.364	68	0.479	1.604 (0.996–2.582)	0.101	1.571 (0.973–2.536)	0.126
T/C–T/T	8	0.057	6	0.042	0.728 (0.246–2.155)	0.812	0.746 (0.251–2.223)	0.839
C/C–A/A	5	0.036	0	0.000	0.000 (0.000–+ ∞)	0.999	0.000 (0.000–+ ∞)	0.999
C/C–A/T	23	0.164	20	0.141	0.834 (0.435–1.599)	0.827	0.791 (0.410–1.526)	0.734
C/C–T/T	10	0.071	3	0.021	0.281 (0.076–1.042)	0.113	0.249 (0.066–0.936)	0.078
**c.47 C > T (p.Val16Ala)**—***SOD2* (rs4880) AND c.660 T > C**—***GPX4* (rs713041)**								
T/T–T/T	3	0.021	5	0.035	1.667 (0.391–7.111)	0.740	1.710 (0.398–7.358)	0.720
T/T–T/C	14	0.100	19	0.134	1.390 (0.668–2.896)	0.614	1.510 (90.718–3.174)	0.2477
T/T–C/C	11	0.079	12	0.085	1.083 (0.461–2.542)	0.979	1.146 (0.485–2.709)	0.940
T/C–T/T	12	0.086	23	0.162	2.062 (0.982–4.326)	0.109	1.959 (0.929–4.133)	0.148
T/C–T/C	34	0.243	43	0.303	1.354 (0.800–2.293)	0.451	1.384 (0.814–2.353)	0.407
T/C–C/C	28	0.200	17	0.120	0.544 (0.283–1.047)	0.131	0.539 (0.279–1.041)	0.128
C/C–T/T	3	0.021	3	0.021	0.986 (0.196–4.968)	0.999	1.025 (0.201–5.213)	0.999
C/C–T/C	17	0.121	10	0.070	0.548 (0.242–1.243)	0.278	0.505 (0.220–1.157)	0.201
C/C–C/C	18	0.129	10	0.070	0.513 (0.228–1.156)	0.203	0.487 (0.215–1.105)	0.163
**c.47 C > T (p.Val16Ala)**—***SOD2* (rs4880) AND g.117803515 C > T**—***NOS1* (rs1879417)**								
T/T–C/C	6	0.043	13	0.092	2.251 (0.830–6.100)	0.210	2.462 (0.900–6.738)	0.152
T/T–C/T	11	0.079	15	0.106	1.385 (0.613–3.131)	0.680	1.387 (0.611–3.151)	0.680
T/T–T/T	11	0.079	8	0.056	0.700 (0.273–1.796)	0.706	0.787 (0.303–2.044)	0.858
T/C–C/C	12	0.086	16	0.113	1.354 (0.616–2.978)	0.698	1.485 (0.669–3.298)	0.552
T/C–C/T	44	0.314	43	0.303	0.948 (0.572–1.571)	0.973	0.929 (0.558–1.545)	0.950
T/C–T/T	18	0.129	24	0.169	1.379 (0.711–2.671)	0.566	1.312 (0.673–2.556)	0.669
C/C–C/C	5	0.036	6	0.042	1.191 (0.355–3.996)	0.950	1.034 (0.303–3.524)	0.998
C/C–C/T	23	0.164	12	0.085	0.470 (0.224–0.985)	0.090	0.470 (0.223–0.990)	0.092
C/C–T/T	10	0.071	5	0.035	0.474 (0.158–1.425)	0.334	0.428 (0.141–1.302)	0.252
**c.47 C > T (p.Val16Ala)**—***SOD2* (rs4880) AND c.1823 C > T (p.Ser608Leu)**—***NOS2* (rs2297518)**								
T/T–C/C	14	0.100	17	0.120	1.224 (0.579–2.590)	0.838	1.311 (0.615–2.796)	0.733
T/T–C/T	10	0.071	8	0.056	0.776 (0.297–2.028)	0.844	0.806 (0.307–2.118)	0.885
T/T–T/T	4	0.029	11	0.077	2.855 (0.887–9.192)	0.152	3.120 (0.960–10.136)	0.113
**T/C–C/C**	**37**	**0.264**	**67**	**0.472**	**2.487 (1.509–4.099)**	**0.002**^**0.951**^	**2.471 (1.495–4.084)**	**0.002**^**0.949**^
T/C–C/T	21	0.150	13	0.092	0.571 (0.274–1.191)	0.252	0.569 (0.272–1.192)	0.252
*T/C–T/T*	*16*	*0.114*	*3*	*0.021*	*0.167* (*0.048–0.588*)	*0.010*^*0.853*^	*0.164* (*0.046–0.579*)	*0.010*^*0.856*^
C/C–C/C	9	0.064	15	0.106	1.719 (0.726–4.069)	0.388	1.697 (0.713–4.037)	0.410
C/C–C/T	17	0.121	8	0.056	0.432 (0.180–1.036)	0.116	0.414 (0.171–1.000)	0.098
C/C–T/T	12	0.086	0	0.000	0.000 (0.000–+ ∞)	0.999	0.000 (0.000–+ ∞)	0.999
**c.660 T > C**—***GPX4* (rs713041) AND c.-89 A > T**—***CAT* (rs7943316)**								
T/T–A/A	4	0.029	2	0.014	0.486 (0.088–2.695)	0.651	0.646 (0.083–2.600)	0.618
T/T–A/T	13	0.093	26	0.183	2.190 (1.075–4.462)	0.061	2.109 (1.031–4.316)	0.080
T/T–T/T	1	0.007	3	0.021	3.000 (0.308–29.194)	0.570	3.274 (0.333–32.173)	0.523
T/C–A/A	13	0.093	9	0.063	0.661 (0.273–1.600)	0.589	0.705 (0.289–1.718)	0.689
T/C–A/T	43	0.307	57	0.401	1.513 (0.925–2.473)	0.188	1.530 (0.933–2.509)	0.176
T/C–T/T	9	0.064	6	0.042	0.642 (0.222–1.854)	0.655	0.624 (0.215–1.815)	0.624
C/C–A/A	7	0.050	2	0.014	0.271 (0.055–1.330)	0.204	0.264 (0.054–1.305)	0.194
C/C–A/T	41	0.293	35	0.246	0.790 (0.66–1.338)	0.617	0.805 (0.474–1.369)	0.688
C/C–T/T	9	0.064	2	0.014	0.208 (0.044–0.980)	0.092	0.182 (0.038–0.868)	0.065
**c.660 T > C**—***GPX4* (rs713041) AND g.117803515 C > T**—***NOS1* (rs1879417)**								
T/T–C/C	4	0.029	6	0.042	1.500 (0.414–5.434)	0.786	1.593 (0.436–5.819)	0.731
T/T–C/T	8	0.057	19	0.134	2.549 (1.077–6.034)	0.065	1.418 (1.016–5.754)	0.090
T/T–T/T	6	0.043	6	0.042	0.985 (0.310–3.132)	0.999	0.948 (0.296–3.038)	0.995
T/C–C/C	17	0.121	19	0.134	1.118 (0.555–2.252)	0.940	1.136 (0.562–2.299)	0.923
T/C–C/T	32	0.229	32	0.225	0.982 (0.562–1.714)	0.997	0.998 (0.569–1.748)	0.999
T/C–T/T	16	0.114	21	0.148	1.345 (90.670–2.700)	0.646	1.359 (0.674–2.740)	0.629
C/C–C/C	2	0.014	10	0.070	5.227 (1.124–24.306)	0.069	5.791 (1.235–27.156)	0.051
*C/C–C/T*	*38*	*0.271*	*19*	*0.134*	*0.415* (*0.225–0.763*)	*0.010*^*0.816*^	*0.409* (*0.222–0.757*)	*0.010*^*0.826*^
C/C–T/T	17	0.121	10	0.070	0.548 (0.242–1.243)	0.278	0.526 (0.231–1.202)	0.240
**c.660 T > C**—***GPX4* (rs713041) AND c.1823 C > T (p.Ser608Leu)**—***NOS2* (rs2297518)**								
**T/T–C/C**	**7**	**0.050**	**24**	**0.169**	**3.864 (1.607–9.295)**	**0.006**^**0.888**^	**3.648 (1.509–8.818)**	**0.008**^**0.851**^
T/T–C/T	11	0.079	3	0.021	0.253 (0.069–0.928)	0.075	0.248 (0.067–0.915)	0.071
T/T–T/T	0	0.000	4	0.028	4,062,394.411 (0.000–+ ∞)	0.999	5,019,550,116 (0.000–+ ∞)	0.999
**T/C–C/C**	**32**	**0.229**	**52**	**0.366**	**1.950 (1.157–3.286)**	**0.024**^**0.713**^	**2.081 (1.225–3.538)**	**0.014**^**0.795**^
T/C–C/T	15	0.107	15	0.106	0.984 (0.462–2.098)	0.999	0.993 (0.464–2.127)	0.999
*T/C–T/T*	*18*	*0.129*	*5*	*0.035*	*0.247* (*0.089–0.686*)	*0.024*^*0.800*^	*0.222* (*0.079–0.625*)	*0.014*^*0.835*^
C/C–C/C	21	0.150	23	0.162	1.095 (0.575–2.085)	0952	1.089 (0.570–2.081)	0.959
C/C–C/T	22	0.157	11	0.077	0.450 (0.210–0.968)	0.080	0.445 (0.206–0.960)	0.076
C/C–T/T	14	0.100	5	0.035	0.328 (0.115–0.938)	0.075	0.332 (0.116–0.954)	0.080
**g.117803515 C > T**—***NOS1* (rs1879417) AND c.-89 A > T**—***CAT* (rs7943316)**								
C/C–A/A	8	0.057	4	0.028	0.478 (0.141–1.626)	0.418	0.493 (0.144–1.688)	0.452
**C/C–A/T**	**15**	**0.107**	**29**	**0.204**	2.139 (1.091–4.193)	0.053	**2.261 (1.145–4.465)**	**0.038**^**0.673**^
C/C–T/T	0	0.000	2	0.014	4,004,360,205 (0.000–+ ∞)	0.999	3,909,419.356 (0.000–+ ∞)	0.999
C/T–A/A	8	0.057	5	0.035	0.602 (0.192–1.888)	0.621	0.586 (0.186–1.852)	0.594
C/T–A/T	58	0.414	60	0.423	1.034 (0.644–1.661)	0.987	1.017 (0.632–1.638)	0.997
C/T–T/T	12	0.086	5	0.035	0.389 (0.133–1.136)	0.161	0.397 (0.136–1.165)	0.177
T/T–A/A	8	0.057	4	0.028	0.478 (0.141–1.626)	0.418	0.512 (0.150–1.755)	0.492
T/T–A/T	24	0.171	29	0.204	1.240 (0.681–2.259)	0.731	1.233 (0.675–2.254)	0.746
T/T–T/T	7	0.050	4	0.028	0.551 (0.158–1.925)	0.578	0.468 (0.133–1.665)	0.424
**g.117803515 C > T**—***NOS1* (rs1879417) AND c.1823 C > T (p.Ser608Leu)**—***NOS2* (rs2297518)**								
C/C–C/C	17	0.121	20	0.141	1.186 (0.593–2.373)	0.862	1.249 (0.620–2.514)	0.782
C/C–C/T	5	0.036	8	0.056	1.612 (0.514–5.053)	0.655	1.534 (0.486–4.845)	0.715
C/C–T/T	1	0.007	7	0.049	7.207 (0.875–59.367)	0.128	8.481 (1.021–70.485)	0.094
**C/T–C/C**	**35**	**0.250**	**57**	**0.401**	**2.012 (1.209–3.346)**	**0.014**^**0.773**^	**1.992 (1.194–2.323)**	**0.016**^**0.760**^
*C/T–C/T*	*24*	*0.171*	*9*	*0.063*	*0.327* (*0.146–0.732*)	*0.014*^*0.795*^	*0.332* (*0.148–0.746*)	*0.016*^*0.786*^
*C/T–T/T*	*19*	*0.136*	*4*	*0.028*	*0.185* (*0.061–0.558*)	*0.006*^*0.899*^	*0.175* (*0.057–0.532*)	*0.004*^*0.909*^
**T/T–C/C**	**8**	**0.057**	**22**	**0.155**	**3.025 (1.298–7.050)**	**0.020**^**0.746**^	**3.040 (1.299–7.116)**	**0.020**^**0.844**^
T/T–C/T	19	0.136	12	0.085	0.588 (0.274–1.262)	0.316	0.584 (0.271–1.259)	0.311
*T/T–T/T*	*12*	*0.086*	*3*	*0.021*	*0.230* (*0.064–0.834*)	*0.049*^*0.629*^	*0.213* (*0.059–0.779*)	*0.038*^*0.653*^
**c.1823 C > T (p.Ser608Leu)**—***NOS2* (rs2297518) AND c.-89 A > T**—***CAT* (rs7943316)**								
C/C–A/A	14	0.100	7	0.049	0.467 (0.182–1.194)	0.211	0.471 (0.183–1.211)	0.222
**C/C–A/T**	**46**	**0.329**	**82**	**0.577**	**2.793 (1.719–4.536)**	**0.002**^**0.988**^	**2.844 (1.743–4.639)**	**0.002**^**0.990**^
C/C–T/T	0	0.000	0	0.000	0.000 (0.000–+ ∞)	0.999	0.000 (0.000–+ ∞)	0.999
C/T–A/A	7	0.050	4	0.028	0.551 (0.158–1.925)	0.578	0.585 (0.166–2.059)	0.645
C/T–A/T	31	0.221	24	0.169	0.715 (0.395–1.294)	0.464	0.698 (0.384–1.268)	0.418
*C/T–T/T*	*10*	*0.071*	*1*	*0.007*	*0.092* (*0.012–0.730*)	*0.047*^*0.667*^	*0.093* (*0.012–0.737*)	*0.049*^*0.667*^
T/T–A/A	3	0.021	2	0.014	0.652 (0.107–3.965)	0873	0.655 (0.107–4.020)	0.875
T/T–A/T	20	0.143	12	0.085	0.554 (0.260–1.181)	0.236	0.562 (0.262–1.204)	0.257
T/T–T/T	9	0.064	0	0.000	0.000 (0.000–+ ∞)	0.999	0.000 (0.000–+ ∞)	0.999

*p* < 0.05 along with corresponding ORs are bold and in bold (for the genotypes/alleles contributing to the MS development) or in italics (for the genotypes/alleles with a protective effect); *denotes *p*-values with the Bonferroni correction; **OR adjusted for gender. Statistical power (1 − β) for significant comparisons is given in superscripts.

We observed that the T/C–C/C combined genotype of rs4880–rs2297518 SNPs (Crude OR 2.487; 1.509–4.099 95% CI; *p* < 0.01) was related to an increased risk of MS, while the T/C–T/T combined genotype of the same polymorphisms reduced this risk (Crude OR 0.167; 0.048–0.588 95% CI; *p* < 0.05).

In addition, we found that the presence of the C/C–C/T combined genotype of the rs713041–rs1879417 polymorphisms reduced the risk of MS development (Crude OR 0.415; 0.225–0.763 95% CI; *p* < 0.05).

Furthermore, in the case of c.660 T > C—*GPX4* (rs713041) and c.1823 C > T—*NOS2* (rs2297518), the T/T–C/C (Crude OR 3.864; 1.607–9.295 95% CI; *p* < 0.01) and T/C–C/C (Crude OR 1.950; 1.157–3.289 95% CI; *p* < 0.05) combined genotypes were associated with an elevated risk of MS development. On the other hand, T/C–T/T (Crude OR 0.247; 0.089–0.686 95% CI; *p* < 0.05 genotype of the same polymorphisms combination contributed to the reduced risk of MS occurrence.

Interestingly, in the case of rs1879417–rs2297518 SNP‐SNP combination, the C/T–C/T (Crude OR 0.327; 0.146–0.732 95% CI; *p* < 0.05), C/T–T/T (Crude OR 0.185; 0.061–0.558 95% CI; *p* < 0.01) and T/T–T/T (Crude OR 0.230; 0.064–0.834 95% CI; *p* < 0.05) genotypes caused a decrease in the risk of MS, whereas the C/T–C/C (Crude OR 2.012; 1.209–3.346 95% CI; *p* < 0.05) and the T/T–C/C (Crude OR 3.025; 1.298–7.050 95% CI; *p* < 0.05) of the same polymorphisms contributed to MS development.

The presence of the C/C–A/T combined genotype of the rs2297518–rs7943316 SNPs was associated with an increased risk of MS (Crude OR 2.793; 1.719–4.536 95% CI; *p* < 0.01) but the C/T–T/T genotype of the same SNP-SNP combination was linked with a reduction of this risk (Crude OR 0.092; 0.012–0.730 95% CI; *p* < 0.05).

On the other hand, synergy factor (SF) analysis (Table 3) proposed by Mario Cortina-Borja et al.^22^ showed that the synergy was only seen between SNPs of the *NOS1* gene and *NOS2* gene (SF = 0.002, *p* < 0.01). Moreover, SF analysis confirmed that the interaction between *NOS1* and *NOS2* polymorphism genotype shows a protective character.

**Table 3:** Synergy factor analysis.

Genes	Genotypes	Synergy factor^a^	*p-*value^b^	Type interaction^a^
SOD2 × CAT	TT–TT	–	–	–
SOD2 × GPX4	TT–CC	1.18	0.89	Antagonistic
SOD2 × NOS1	TT–TT	0.80	0.84	Antagonistic
SOD2 × NOS2	TT–TT	–	–	–
GPX4 × CAT	CC–TT	0.13	0.26	Synergistic
GPX4 × NOS1	CC–TT	0.18	0.16	Synergistic
GPX4 × NOS2	CC–TT	–	–	–
NOS1-CAT	TT–TT	–	–	–
**NOS1 × NOS2**	**TT–TT**	**0.02**	**< 0.01**	**Synergistic, protectivec**
NOS2 × CAT	TT–TT	–	–	–

*p* < 0.05 s are bold and in bold for the genotypes with a protective effect.

^a^All SF relate to the risk of MS. The cited genotypes were treated as risk factors, unless otherwise stated; the terms, ‘risk’ and ‘protective’ factors, refer to associations, i.e. no causality is implied. Note that synergy (antagonism) between risk factors will produce a SF > 1 (< 1), while synergy (antagonism) between protective factors will give a SF < 1 (> 1).

^b^All *p*-values are before correction for multiple testing, whether or not relevant.

### SNPs of genes encoding enzymes involved in nitrative and oxidative stresses and MS occurrence in the male and female subpopulation

A previous epidemiological study showed that women were exposed to at least a doubled risk of MS when compared to men^10^. Thus, we studied the link between the MS occurrence in male or female groups and all examined polymorphisms.

#### The c.-420–34,221 G > A (rs1879417) SNP of the NOS1 and the risk of MS occurrence in male and female groups

Our findings show that the g.117803515 C > T—*NOS1* SNP was not significantly associated with the risk of MS in both, male and female subpopulations (Table 4).

**Table 4:** Distribution of genotypes and alleles of the g.117803515 C > T—*NOS1* (rs1879417), c.1823 C > T (p.Ser608Leu)—*NOS2* (rs2297518), c.47 C > T—*SOD2* (rs4880), c.-89 A > T—*CAT* (rs7943316), c.660 T > C—*GPX4* (rs713041) and Ors with 95% Cis in men and women with MS.

Genotype/allele	Men (n = 118)				Women (n = 164)			
	Control (n = 51)	MS (n = 67)	Crude OR (95% CI)*	*p*	Control (n = 89)	MS (n = 75)	Crude OR (95% CI)*	*p*
	N (freq.)	N (freq.)			N (freq.)	N (freq.)		
**c.-420-34221G > A**—***NOS1* (rs1879417)**								
G/G	6 (0.118)	14 (0.209)	1.981 (0.703–5.580)	0.196	17 (0.191)	21 (0.280)	1.647 (0.793–3.419)	0.181
G/A	27 (0.529)	37 (0.552)	1.096 (0.528–2.277)	0.805	51 (0.573)	33 (0.440)	0.585 (0.315–1.088)	0.090
A/A	18 (0.353)	16 (0.239)	0.575 (0.258–1.284)	0.177	21 (0.236)	21 (0.280)	1.259 (0.624–2.542)	0.520
	χ^2^ = 2.786; *p* = 0.095				χ^2^ = 0.169; *p* = 0.681			
G	39 (0.382)	65 (0.485)	1.605 (0.913–2.819)	0.100	85 (0.478)	75 (0.500)	1.097 (0.706–1.704)	0.681
A	63 (0.618)	69 (0.515)	0.623 (0.355–1.095)	0.100	93 (0.522)	75 (0.500)	0.912 (0.587–1.417)	0.681
**c.1823C > T (p.Ser608Leu)**—***NOS2* (rs2297518)**								
C/C	**18 (0.353)**	**48 (0.716)**	**4.632 (2.118–10.127)**	**< 0.001**^**0.978**^	**42 (0.472)**	**51 (0.680)**	**2.378 (1.255–4.506)**	**0.008**^**0.767**^
C/T	18 (0.353)	14 (0.209)	0.484 (0.213–1.102)	0.084	30 (0.337)	15 (0.200)	0.402 (0.240–1.007)	0.052
T/T	*15* (*0.294*)	*5* (*0.075*)	*0.194* (*0.065–0.577*)	*0.003*^*0.855*^	17 (0.191)	9 (0.120)	0.578 (0.241–1.384)	0.218
	χ^2^ = 17.534; *p* < 0.001				χ^2^ = 5.804; *p* = 0.016			
C	**54 (0.529)**	**110 (0.821)**	**2.958 (1.716–5.100)**	** < 0.001**^**0.774**^	**114 (0.640)**	**117 (0.780)**	**1.683 (1.090–2.598)**	**0.019**^**0.326**^
T	*48* (*0.471*)	*24* (*0.179*)	*0.338* (*0.196–0.583*)	< *0.001*^*0.774*^	*64* (*0.360*)	*33* (*0.220*)	*0.594* (*0.385–0.917*)	*0.019*^*0.327*^
**c.47C > T (p.Val16Ala)**—***SOD2* (rs4880)**								
*C/C*	16 (0.314)	15 (0.224)	0.631 (0.277–1.439)	0.274	*22* (*0.247*)	*8* (*0.107*)	*0.364* (*0.151–0.840*)	*0.024*^*0.640*^
T/C	27 (0.529)	41 (0.612)	1.402 (0.671–2.930)	0.369	47 (0.528)	42 (0.560)	1.137 (0.613–2.109)	0.683
T/T	8 (0.157)	11 (0.164)	1.056 (0.391–2.852)	0.915	20 (0.225)	25 (0.333)	1.725 (0.864–3.444)	0.122
	χ^2^ = 0.663; *p* = 0.415				χ^2^ = 5.707; *p* = 0.017			
*C*	59 (0.578)	71 (0.530)	0.790 (0.446–1.397)	0.417	*91* (*0.511*)	*58* (*0.387*)	*0.565* (*0.350–0.911*)	*0.019*^*0.434*^
**T**	43 (0.422)	63 (0.470)	1.267 (0.716–2.242)	0.417	**87 (0.489)**	**92 (0.613)**	**1.769 (1.097–2.853)**	**0.019**^**0.434**^
**c.-89 A > T**—***CAT* (rs7943316)**								
A/A	7 (0.137)	6 (0.090)	0.618 (0.194–1.967)	0.415	17 (0.191)	7 (0.093)	0.436 (0.170–1.117)	0.084
**A/T**	**34 (0.667)**	**56 (0.836)**	**2.545 (1.066–6.075)**	**0.035**^**0.553**^	63 (0.708)	62 (0.827)	1.968 (0.927–4.177)	0.078
T/T	10 (0.196)	5 (0.075)	0.331 (0.105–1.038)	0.058	9 (0.101)	6 (0.080)	0.773 (0.262–2.281)	0.641
	χ^2^ = 0.667; *p* = 0.414				χ^2^ = 1.021; *p* = 0.312			
A	48 (0.471)	68 (0.507)	1.368 (0.642–2.916)	0.417	97 (0.545)	76 (0.507)	0.719 (0.378–1.369)	0.316
T	54 (0.529)	66 (0.493)	0.713 (0.343–1.558)	0.417	81 (0.455)	74 (0.493)	1.390 (0.731–2.644)	0.316
**c.660 T > C**—***GPX4* (rs713041)**								
C/C	*23* (*0.451*)	*17* (*0.254*)	*0.414* (*0.190–0.902*)	*0.026*^*0.605*^	34 (0.382)	22 (0.293)	0.671 (0.349–1.294)	0.234
T/C	21 (0.412)	33 (0.493)	1.387 (0.665–2.892)	0.384	44 (0.494)	39 (0.520)	1.108 (0.599–2.049)	0.744
T/T	7 (0.137)	17 (0.254)	2.137 (0.811–5.632)	0.124	11 (0.124)	14 (0.187)	1.627 (0.690–3.837)	0.266
	χ^2^ = 5.548; *p* = 0.019				χ^2^ = 2.052; *p* = 0.152			
C	*67* (*0.657*)	*67* (*0.500*)	*0.537* (*0.316–0.913*)	*0.022*^*0.375*^	112 (0.629)	83 (0.553)	0.716 (0.453–1.134)	0.155
T	**35 (0.343)**	**67 (0.500)**	**1.862 (1.095–3.167)**	**0.022**^**0.375**^	66 (0.371)	67 (0.447)	1.396 (0.882–2.209)	0.155

*p* < 0.05 along with corresponding ORs are bold and in bold (for the genotypes/alleles contributing to the MS development) or in italics (for the genotypes/alleles with a protective effect); *OR adjusted for gender. Statistical power (1 − β) for significant comparisons is given in superscripts.

#### The c.1823 C > T (rs2297518) SNP of the NOS2 and the risk of MS occurrence in male and female groups

We also detected that the C/C homozygote (Crude OR 4.632; 2.118–10.127 95% CI; *p* < 0.001; Crude OR 2.378; 1.255–4.506 95% CI; *p* < 0.01, respectively) and the C allele (Crude OR 2.958; 1.716–5.100 95% CI; *p* < 0.001; Crude OR 1.683; 1.090–2.598 95% CI; *p* < 0.05) of the c.1832 C > T—*NOS2* SNP were associated with an elevated risk of MS in both, male and female groups. On the other hand, the T allele (Crude OR 0.338; 0.196–0.583 95% CI; *p* < 0.001; Crude OR 0.594; 0.385–0,917 95% CI; *p* < 0.05) of the same polymorphism was associated with a reduced of the disease in male and female subpopulations. Whereas, the T/T homozygote (Crude OR 0.194; 0.065–0.577 95% CI; *p* < 0.01) of the same polymorphism reduced the MS risk in only male subpopulation (Table 4).

#### The c.47 C > T (rs4880) SNP of the SOD2 and the risk of MS occurrence in male and female groups

We found that the C/C genotype and the C allele of the c.47 C > T—*SOD2* SNP were association with a reduced risk of MS development in female subpopulation (Crude OR 0.364; 0.151–0.840 95% CI; *p* < 0.05; Crude OR 0.565; 0.350–0.911 95% CI; *p* < 0.05, respectively), while in male subpopulation we did not observe this correlation.

On the other hand, the T allele of the same polymorphism was linked to a higher risk in the female subpopulation (Crude OR 1.769; 1.097–2.853 95% CI; *p* < 0.05, while this dependence was not observed in males (Table 4).

#### The c.-89 A > T (rs7943316) SNP of the CAT and the risk of MS occurrence in male and female groups

In the case of the c.-89A > T—*CAT* polymorphism, the heterozygote (Crude OR 2.545; 1.066–6.075 95% CI; *p* < 0.05) was associated with an elevated risk of MS in the male subpopulation, while in the female subpopulation we did not observe this correlation (Table 4).

#### The c.660 T > C (rs713041) SNP of the GPX4 and the risk of MS occurrence in male and female groups

As shown in Table 4, the C/C genotype and the C allele of the c.660 T > C—*GPX4* SNP were linked with a decreased frequency of MS occurrence in the male group (Crude OR 0.414; 0.190–0.902; 95% CI; *p* < 0.05; Crude OR 0.537; 95% CI; *p* < 0.05, respectively), while this dependence was not observed in females. Moreover, the T allele was associated with an increased risk of MS occurrence in the only male subpopulation (Crude OR 1.862; 1.095–3.167 95% CI; *p* < 0.05).

## Discussion

MS is a multifactorial immune-mediated disease, most probably caused by the complex bidirectional interaction of environment-gene. Among the numerous agents that favor the development of MS, attention is paid to the genetic factors, mainly including disorders of genes associated with human leukocyte antigens (HLA) and environmental factors, such as infectious mononucleosis, smoking, adolescent obesity, and vitamin D deficiency^23^. However, despite the identification of multiple risk factors for MS, the pathogenesis of the disease still remains unclear, and previous studies have focused only on the primary role of inflammation in the pathogenesis of MS. Nevertheless, there are growing new reports that point out the important role of both nitrative and oxidative stresses in MS development^15,24,25^. However, it should be noted that RNS and ROS exhibit a dual nature, and their neurotoxicity is only associated with their high levels.

Nitric oxide (NO) is considered as one of the main RNS and at a physiological level in the CNS, regulates many functions, including synaptic plasticity, the sleep–wake cycle, and hormone secretion. NO, as a signaling molecule, primarily influences an increase in activity with soluble guanylyl cyclase (sGC), thus leading to an enhancement in the production of cyclic guanosine monophosphate (cGMP). In turn, high intracellular levels of cGMP may impact synaptic plasticity, smooth-muscle relaxation, neurosecretion, and neurotransmission. Furthermore, NO is also involved in the regulation of pathways that promote cell survival, such as the Akt kinase pathway and cyclic AMP-transcription factor binding protein (CREB). Interestingly, NO as a signaling molecule also stimulates various areas of the brain to secrete neurotransmitters, e.g. acetylcholine in the nucleus accumbens, as well as dopamine and serotonin in the medial preoptic area^26^. On the other hand, to data, the results showed that excessive nitrative and oxidative stress, resulting from the genetic predisposition and environmental factors, which may lead to increased production of RNS and ROS, as well as may induce neuroinflammation, observed in course of neurodegenerative diseases (e.g. Alzheimer’s disease, Parkinson’s disease, depression, and MS). Therefore, the initiating factor of MS development seems to be NO, generated by iNOS (inducible nitric oxide synthase). In turn, high iNOS transcription is induced by IFN-β (interferon β) and TNF-α (tumor necrosis factor α) in astrocytes, microglia, and macrophages. Consequently, the activity of NO and pro-inflammatory cytokines contributes to the myelin damage^16–18^. The myelin peptides from the breakdown of the myelin sheath of nerve cells induce migration of neutrophils to the site of inflammation in the CNS. In addition, activated neutrophils, by increasing the secretion of matrix metalloproteinases, mediate the degradation of tight junctions between endothelial cells, which may consequently lead to the disruption of the BBB and the massive migration of various immune cells to the CNS. The transmission of neutrophils through the damaged endothelium to the CNS enhances their pro-inflammatory phenotype and increases the degranulation process and the secretion of pro-inflammatory mediators, such as TNF-α, IL-1β (interleukin 1β), and IL-6. Moreover, activation of neutrophils induces the activity of pro-oxidative enzymes, including NADPH oxidase (NOX) and myeloperoxidase (MPO). NOX generates the formation of superoxide anion radicals, which are then converted by SOD to H_2_O_2_. In contrast, MPO converts H_2_O_2_ into hypochlorous acid and an oxygen burst occurs. The uncontrolled ejection of ROS associated with increased activation of neutrophils leads to the activation of microglia cells and further damage to the myelin sheath and thus leads to the progression of MS^27^.

In the case of nerve cells, low levels of ROS are essential for regulating their normal growth and development and are involved in long-term potentiation (LTP) through glutamate-dependent mechanisms. LTP, as a neuronal mechanism located in the CNS, is the basis for memory formation and learning and increases the intensity of synaptic conduction^28,29^. Moreover, both RNS and ROS are important physiological mediators of synaptic plasticity and neuronal signaling. Previous studies showed that ROS at a low level can regulate the activity of signaling molecules, receptors, and channels related to synaptic plasticity, including N-methyl-d-aspartate (NMDA) receptors, calcium (Ca^2+^) channels, potassium (K^+^) channels, Ca^2+^/calmodulin kinases II (CaMKII), extracellular signal-regulated kinase (ERK) and cyclic adenosine monophosphate (cAMP) response element-binding protein (CREB) in the hippocampus^11^. On the other hand, when ROS accumulate excessively in the brain, they can become detrimental to the function of neurons, leading to the development of neurodegenerative disease. A high level of ROS leads to oxidative modifications of cellular proteins, lipids, and DNA, causing cell dysfunction and may modulate survival signaling pathways^11^.

As above mentioned, previous data suggest that genetic factors can be one of the factors, play a crucial role in the MS course. Genome-wide association studies (GWAS), based on a high-density SNP genotyping array, have identified many chromosomal regions/gene *loci* associated with MS risk. A recent GWAS study on a large scale conducted by International Multiple Sclerosis Genetics Consortium (IMSGC), covering more than 100,000 people provided solid evidence for linking 200 autosomal loci with MS risk. Actually, MS can be associated with severely susceptibility *loci*, including chromosome 6p21 (three serological alleles of the HLA, encoded in the major histocompatibility complex), 10p15.1 [encoding the interleukin‐2 receptor (IL‐2RA)] and 5p13.2 [encoding the interleukin‐7 receptor (IL‐7RA)]^30^, as well as 12p13.31 [encoding the C-type lectin-like 1 (CLECL1)] 17q21.32 [encoding the EF-hand calcium-binding domain 13 (EFCAB13)]^31,32^. These findings confirm a heterogeneous and complex genetic character of MS and the genotyping approach will ensure a better understanding and identification of basic genetic defects.

Previous studies have shown that SNPs, having a functional effect on the course of oxidative stress pathways, can be a risk factor for different diseases, including neurodegenerative disease^33–35^. However, despite abundant evidence suggesting a significant role of nitrative and oxidative stress in the development of MS, the literature review shows that no studies point to the association of polymorphisms located in genes involved in these pathways and the modulation of MS occurrence risk. Therefore, the presented study was undertaken to the identification of the potential association of five SNPs in nitrative and oxidative stress-related genes: *NOS1* (g.117803515 C > T; rs1879417) and *NOS2* (c.1823 C > T; rs2297518) as well as *SOD2* (c.47 C > T; rs4880), *CAT* (c.-89 A > T; rs7943316), *GPX4* (c.660 T > C; rs713041), and the MS occurrence. To the best of our knowledge, we were the first who detect that analyzed polymorphisms of the genes associated with oxidative stress may modulate the risk of MS occurrence.

As noted above, the development of MS is associated not only with impaired antioxidant defense but also with excessive production of RNS as well as ROS. Therefore, our research also included the assessment of the impact of polymorphisms located in the *NOS1* and *NOS2* genes. NOS1 and NOS2 belong to the NO synthase family, members of which converse the L-arginine to NO. So far, this family includes three isoforms, i.e. the neuronal type I (NOS1) and the endothelial type (eNOS), which are constitutively expressed and regulated by calmodulin and Ca^2+^, and the iNOS (also known as NOS2). A high-level expression of NOS1 is characteristic of nervous tissue, while iNOS produces large quantities of NO upon stimulation by pro-inflammatory cytokines^29,36^. Interestingly, according to data provided by NCBI dbSNP, 56,540 SNPs in *NOS1* have been registered, while *NOS2* has 16,601 various polymorphisms. Numerous polymorphisms, occurring within this gene [especially g.117803515 C > T—*NOS1* (rs1879417) and c.1823C > T—*NOS2* (rs2297518)], play an essential role in the development mechanism of various diseases, including Fanconi anemia, depression, stroke, gastric, and urinary bladder cancer^35,37–40^. The first analyzed polymorphism in our study is g.117803515 C > T—*NOS1* (rs1879417) localized in the intron on chromosome 12, at position 12q24.22. Intron location of polymorphism can affect the mRNA/protein splicing process, leading to the formation of different protein isoforms^41,42^. Our study did not show any association between g.117803515 C > T SNP in the *NOS1* and the MS occurrence. However, in the case of c.1823 C > T (rs2297518) polymorphism of the *NOS2* gene, we detected that the C/C genotype and the C allele were associated with increased MS risk, whereas the heterozygote and T/T homozygote, as well as T allele, reduced this risk. The c.1823 C > T—(rs2297518) SNP is located in *NOS2* gene in the 17q11.2–q12 region. The rs2297518 polymorphism leads to the substitution at the amino acid position 608 from serine (Ser) to leucine (Leu) (p.Ser608Leu). Dhillon et al. found that this substitution is correlated with the increase of NOS2 activity, and the A allele confers higher NO generation^43^. However, our findings showed an association of the C/C genotype and C allele with the occurrence of MS, while the T/T homozygote, heterozygote, and T allele were associated with a reduced risk of MS. Moreover, additional analysis, including gender, showed that this polymorphism is important both in men’s and women’s subpopulations.

Nitrative stress inevitably accompanies oxidative stress, thus, in our study we also analysed polymorphisms located in genes, encoding antioxidant enzymes. We found a significant link between c.47 C > T—*SOD2* (rs4880) polymorphism and the MS occurrence. *SOD2* is a gene located on the long arm of chromosome 6 at position 6q25, encoding mitochondrial manganese superoxide dismutase (MnSOD). SOD2 is an essential element of cell defense against the toxic effects of free oxygen radicals. The increased activity of this enzyme leads to an enhancement in the level of H_2_O_2_, which is then removed by CAT. Therefore, numerous polymorphisms, that occur within this gene [especially c.47C > T—*SOD2* (rs4880)], play an essential role in the development mechanism of various diseases, including cancers (breast, prostate, bladder, and cervical cancers), depression, coronary artery disease, and type 1 diabetes^35,44–48^. According to data provided by NCBI dbSNP, 39,247 SNPs in this gene have been registered. The potential of the rs4880 polymorphism as a diagnostic marker is extremely important, due to the fact that its appearance involves functional changes to the *SOD2* gene. This SNP is located in exon 2 and substitutes a C > T at position 47 in the coding sequence, leading to the change of alanine (Ala) to valine (Val) at the amino acid position 16 (p.Val16Ala). Previous results suggest that the T allele of this polymorphism is associated with decreased expression and production of an unstable mRNA, which affects the reduction of its antioxidant potential in mitochondria^49,50^. On the other hand, the Val variant was associated with a 30–40% increase in SOD2 activity in mitochondria^51^. Similarly, obtained results confirmed that the C/C genotype reduced the MS risk in a Polish population. Moreover, our results assert epidemiological data that women are more exposed to MS development than men^52^. The additional analysis, including gender, showed that this polymorphism is important in the women's subpopulation, while in the subpopulation of men there were no significant differences in the presence of genotypes of this polymorphism between the group of patients with MS and the control group. However, MS is not the only autoimmune disease characterized by a higher incidence in women than in men. A similar association is also observed for diseases, such as rheumatoid arthritis, systemic lupus erythematosus, and thyroiditis^53^. Although the causes of this gender difference are unknown, we may speculate that these variances may be the result of the decline in the activity or lower concentration of antioxidant enzymes, which may contribute to increased RNS and ROS accumulation in men and women subpopulations. These differences between men and women subpopulation in antioxidant properties may be due to an estrogen. An estrogen acts as an antioxidant by scavenging free radicals due to the presence of a phenolic hydroxyl group. Animal studies showed that after castration, oxidative stress was higher in female rats compared to control females, while no significant difference was observed in male after castration. On the other hand, estrogen helps to increase the production of mitochondrial ROS, which is involved in cell signaling pathways. This discrepancy was explained by further molecular analyzes, which confirmed that estrogen selectively influences the expression level of antioxidant enzymes, including SOD and GPX^54^. Interestingly,studies on neurodegenerative diseases has shown that testosterone acts as an oxidative stressor in neurons and, in addition, may enhance oxidative damage to these cells^55^. Previous studies showd that testosterone may increase intracellular calcium release in neuron, leading to an intensification of ROS generation and therefore may contribute to neurodegeneration^56^.

The next analyzed polymorphism is localized in the *CAT* gene. This gene is located in the region 11p13 and encodes an antioxidant enzyme, which is involved in the conversion of the hydrogen peroxide into water and oxygen, and thus protects cells against damage, caused by ROS^57^. According to the NCBI dbSNP database, 12,433 polymorphisms have been identified so far. The rs7943316 SNP is located in the *CAT* promoter adjacent to the start site and substitutes A for T at position − 89. The promoter is a necessary element required for the initiation of the transcription process^57^. Moreover, any change in the sequence of this region can cause a gene function termination or alteration. Therefore, SNP in promotor can increase or reduce expression on mRNA level and consequently influence the protein expression^58,59^. Previous studies confirmed that c.-89 A > T—*CAT* (rs7943316) polymorphism was associated with the development of various diseases, including depression, infertility, keratoconus, thyroid, and hepatocellular carcinoma^35,59–61^. In the case of c.-89 A > T—*CAT* (rs7943316) polymorphism, we found that heterozygote was characterized by an increased risk of MS. Moreover, similarly to SNP of *SOD2*, we found differences in the group of women and men. However, for the polymorphism located in the *CAT* gene, we demonstrated a significant effect of the studied SNP on the risk of developing MS in the group of men, which has not been reported in women.

We also found that c.660 T > C—*GPX4* (rs713041) SNP may contribute to MS development. The c.660 T > A polymorphism is located on the short arm of chromosome 19 at position 19p13.3 in the 3′UTR region (exon 7). *GPX4* encodes antioxidant selenocysteine, which catalyzes H_2_O_2_, organic hydroperoxides, and lipid hydroperoxides reduction via means of glutathione oxidation^62–64^. The NCBI dbSNP database declares 2968 identified polymorphisms located in the gene encoding *GPX4*, including rs713041. This polymorphism is a silent mutation, which changes the amino acid from leucine (Leu) to Leu at position 220 (p.Leu220)^65^. Although there is no amino acid change, it is responsible for the appearance of the affinity alternation of the selenocysteine insertion machinery for its SECIS (selenocysteine insertion sequence) element and thus can modulate the GPX4 synthesis^65^. Moreover, Bermano et. al. (2007) found that the C variant was stronger than the T variant at driving the biosynthesis of a GPX4 reporter^66^. Accordingly, our results indicated that the C/C genotype and the C allele were associated with a reduced risk of MS development, while the T/T genotype and the T allele were positively associated with this risk. Moreover, we were the first who demonstrate that the T/T genotype and the T allele of c.660 T > C—*GPx4* (rs713041) SNP decreased the risk of MS occurrence, while the C allele increased this risk but in the only male subpopulation. In the female subpopulation, we observed no impact of this polymorphism on MS risk.

What is especially noteworthy is that, our data indicate for the first time that MS susceptibility may be modulated not only by single locus with genetic main effects, but also by epistatic (gene–gene) interactions in oxidative stress-related genes studied in this paper. We found a strong association between a number of two-gene combinations and the increased/decreased risk of a MS: rs4880–rs2297518, rs713041–rs2297518, rs1879417–rs7943316, rs1879417–rs2297518, rs rs2297518–rs7943316, rs713041–rs1879417. However, SF analysis confirmed that the protective synergy was only seen between SNPs of the *NOS1* gene and *NOS2* gene.

We have as first demonstrated that the selected polymorphisms, localized in the genes associated with nitrative and oxidative stress may have an impact on the risk of MS occurrence. However, we are aware that the presented study has some limitations. First of all, we want to clearly emphasize that our case–control study is preliminary, limited to a single population and relatively small sample size, which in turn gives the possibility that the results may not be duplicated in other populations (the statistical power for some genotypes/alleles was below 80%). Another limitation that should not be forgotten is the ethnic origin of the study participants. Therefore, the results obtained in our study cannot be freely extrapolated to other ethnic groups. The above limitations result from the specificity of the target material and its limited availability. Moreover, it seems interesting in the future to perform additional analysis of the genotype distribution of the studied polymorphisms, taking into account the analysis of the level of gene expression. To summarize, we want to clearly emphasize that our study is preliminary and suggests the need for further research on more number of patients. Therefore, the obtained results should be treated with caution.

## Materials and methods

### Subjects

The study was carried out on a group of 142 patients suffering from MS, hospitalized at the Neurological Rehabilitation Division III General Hospital in Lodz, Poland, and 140 healthy volunteers from which blood samples were collected at Laboratory Diagnostics Center in Lodz, Poland. All participants of the control and MS groups were native Poles from central Poland (not related), randomly selected without replacement sampling. Moreover, the control group was matched by age and gender. The characteristics of the patients and volunteers are presented in Table 5.

**Table 5:** Characteristics of study participants.

Characteristics	Controls (n = 140)	MS (n = 142)
**Gender**		
Male	51 (36%)	67 (47%)
Female	89 (64%)	75 (53%)
Age (mean ± SD)	45 ± 12	53 ± 14
**BMI (kg/m**^**2**^**)**		
Underweight (≤ 18.5)	22.2 ± 2.45	19.45 ± 4.67
Normal weight (18.5–24.9)		
Overweight (25–29.9)		
Obesity (≥ 30)		
Mean disease duration (years)	N/A	14.5 ± 8.1
**EDSS**		
From normal to mid disability (1.0–4.5)	N/A	5.5. ± 1.9
From moderate to severe disability (5.0–9.5)		
Death due to MS (10.0)		
**MMSE**		
Normal (25–30)	N/A	23.79 ± 6
Mild impairment (21–24)		
Moderate impairment (10–20)		
Severe impairment (≤ 9)		
**BDI**		
Normal (≤ 9)	N/A	10 ± 4
Minimal depressive symptomatology (10–15)		
Mild depression (16–31)		
Moderate depression (32–47)		
Severe depression (≥ 47)		
**GDS**		
Normal (0–4)	N/A	7.5 ± 4.56
Mild depression (5–8)		
Moderate depression (9–11)		
Severe depression (12–15)		

*N/A* not applicable.

Enrolled patients were observed for one year and diagnosed according to the revised McDonald criteria, which were established by Lublin et al.^67^, and were not taking the disease-modifying therapies for at least a year.

The functional status of each MS patient was examined using the Expanded Disability Status Scale (EDSS). For the mild cognitive impairment detection, the Mini-Mental State Examination (MMSE) was used, while depression status was assessed using the Beck Depression Inventory (BDI) and Geriatric Depression Scale (GDS).

All included healthy volunteers were not taking any medications and supplements, as well as they had never been diagnosed with any neurological, metabolic, and hormonal illnesses, or any inflammatory diseases and other chronic disorders. Furthermore, each volunteer enrolled in the study was screened (including the full panel of hematological and biochemical tests) to exclude any possible medical conditions.

Participation was voluntary in this presented study. All subjects were informed about the details of the study and confidentiality as well as assured of their voluntary participation in the experiment. All participants gave their written informed consent before participation. During hospitalization, all patients were treated according to MS rehabilitation standards. All procedures were carried out according to the Helsinki Declaration and were approved by the Ethics Committee of the Medical University of Lodz, Poland No. 15/KBBN-UŁ/II/2016.

### Selection of SNPs

The selection of studied polymorphisms of five crucial oxidative/nitrative stress genes done based on the data contained in the public domain of the database for SNPs of the National Center for Biotechnology Information (NCBI dbSNP), available at http://www.ncbi.nlm.nih.gov/snp (Bethesda, Montgomery County, MD, USA; accessed on 01 January 2022).

The selection criteria regarding SNPs were as follows: (1) their minor allele frequency (MAF) had to be larger than 0.05 (submitter population ID: HapMap‐CEU), and (2) they had to be localized either in the coding or regulatory region of the genes as well as may have functional meaning for transcription and protein function (Table 6).

**Table 6:** Basic information of studied SNPs.

Gene	Chromosome location	SNPs	SNP ID rs number	Region	Function	MAF in European population
*SOD2*	6	c.47 C > T (p.Val16Ala)	rs4880	Exon	This polymorphism causes a substitution of Val to Ala in codon 16 at position 9 and in consequence, leads to functional modulation of protein; the 16Ala variant with α-helical structure shows normal transportation of the enzyme into the mitochondria, while the 16Val-containing precursor, which has a β-sheet conformation has 30–40% reduced enzymatic activity	C: 0.498
*CAT*	11	c.-89 A > T	rs7943316	5′ UTR	The T/T genotype of this SNP is associated with a reduction in catalase activity compared to the A/A and A/T genotypes^68^	A: 0.349
*GPX4*	19	c.660 T > C (p.Leu220 =)	rs713041	3′ UTR	This polymorphism causes the change of the amino acid at position 220 from leucine to leucine (p.Leu220) in a region of the *GPX4* corresponding to the 3′-untranslated region (3′-UTR) of the mRNA and alters the protein binding to the 3′-UTR and reporter gene activity, which in turn modulates the ability of the GPX4 3′UTR both to promote GPX4 synthesis and to compete for components of the selenoprotein synthetic machinery, and thus influence the synthesis of a range of selenoproteins. Summarizing, this SNP is involved in the modulation of the GPX4 synthesis by altering the affinity of the selenocysteine insertion machinery for its SECIS element^65,69^	T: 0.452
*NOS1* (n*NOS*)	12	g.117803515 C > T	rs1879417	Intron	SNPs, which are localised in intron can affect the mRNA/protein splicing process, resulting in the formation of different isoforms of a protein^41,42^	C: 0.459
*NOS2* (*iNOS*)	17	c.1823 C > T (p.Ser608Leu)	rs2297518	Exon	This polymorphism causes an amino acid substitution from serine to leucine which increases iNOS activity (alters iNOS protein function) and conferees higher NO production based on the A-allele^43^	T: 0.198

### DNA extraction and genotyping

Genomic DNA was isolated from venous blood collected in CPDA-1 (citrate phosphate dextrose adenine-1) tubes (Sarstedt^®^, Nümbrecht, Germany), in the morning (8–9 a.m.) in fasting status, using the Blood Mini Kit protocol (A&A Biotechnology, Gdynia, Poland) according to the manufacturer’s instructions. Blood samples were collected from the patients suffering from MS before the commencement of the rehabilitation therapy. The purity of the DNA samples was measured spectrophotometrically using a Bio-Tek Synergy HT Microplate Reader (Bio-Tek Instruments, Winooski, VT, USA) by calculating the ratio between absorbance at 260 and 280 nm; after that, the samples were stored at − 20 °C until further analysis.

The chosen polymorphisms were genotyped using the Taq-Man^®^ SNP Genotyping Assays (assay ID: C_8709053_10—rs4880; C_1883210_10—rs7943316; C_2561693_20—rs713041; C_11754652_10—rs1879417; C_11889257_10—rs2297518; Thermo Fisher Scientific, Waltham, MA, USA) and TaqMan Universal Master Mix II, no UNG (Thermo Fisher Scientific, Waltham, MA, USA), according to the manufacturer’s recommendations. The cycling conditions for amplifying PCR products are presented in Table 7.

**Table 7:** Thermal cycling conditions.

Thermal cycling conditions			
Step	Temperature (°C)	Time (s)	Cycle number
AmpliTaq Gold^®^ enzyme activation	95	600	1
Denaturation	95	15	40
Elongation	60	60	

**Figure 1:**
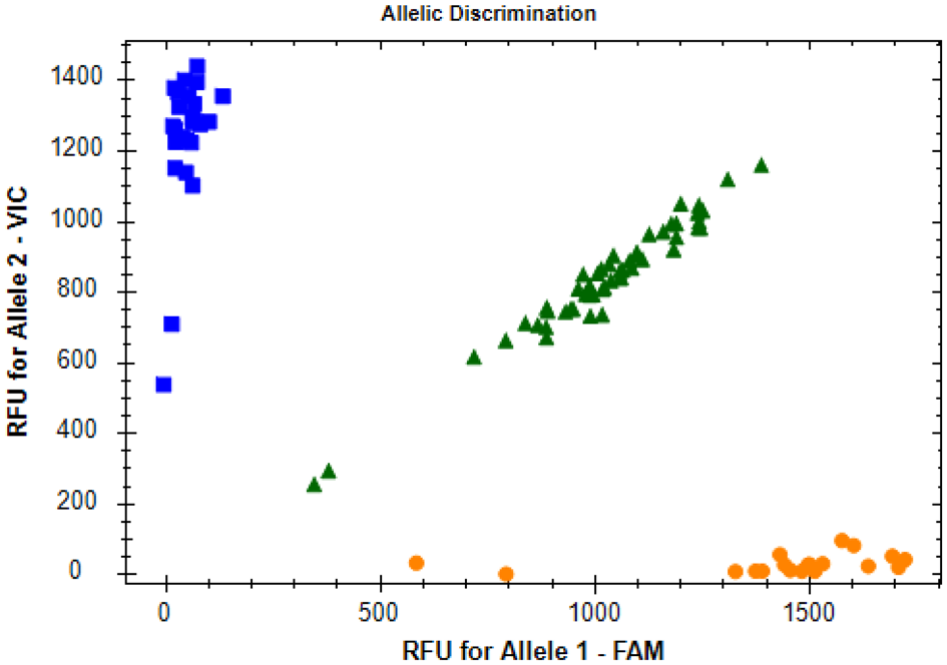
Allelic discrimination X–Y scatter-plot of the c.47 C > T (p.Val16Ala) SNP (rs4880) of the *SOD2*. The TaqMan^®^ SNP Genotyping Assay (ID: C_8709053_10) was used for genotyping of this polymorphism. The X-axis represents the relative fluorescent emission for the C allele-specific probe labeled with 6-carboxyfluorescein (FAM), and the Y-axis represents the emission for the T allele-specific probe labeled with 2′-chloro-7′-phenyl-1,4-dichloro-6-carboxyfluorescein (VIC). Circles: homozygous C/C; squares: homozygous T/T; triangles: heterozygous T/C.

Real-time PCRs were carried out in the thermal cycler CFX96™ Real-Time PCR Detection System (BIO-RAD, Hercules, CA, USA) and genotypes were automatically analyzed based on fluorescent emission data depicted in the X–Y scatter-plot of the CFX Manager™ Software (BIO-RAD, Hercules, CA, USA). A representative allelic discrimination X–Y scatter-plot of the c.47 C > T (p.Val16Ala) SNP (rs4880) of the *SOD2* is presented in Fig. 1.

### Statistical analysis

The analysis of collected results was performed in Statistica 12 (Statsoft, Tulsa, OK, USA) and SigmaPlot 11.0 (Systat Software Inc., San Jose, CA, USA). Additionally, G*Power 3.1 (Heinrich-Heine-Universität, Düsseldorf, Germany) used for power analysis^70^. Hardy–Weinberg equilibrium was checked using χ^2^ test to compare the observed genotype frequencies with the expected frequencies among the case and control subjects. The χ^2^ analysis was also used to test the significance of the differences between distributions of genotypes and alleles in MS patients and controls. The unconditional multiple logistic regression model (codominant, dominant and recessive models) had been used to obtain the ORs and its corresponding 95% CI with *p*-values for MS risk. Additionally, *p*-values obtained for gene–gene analyses were corrected for multiple testing using the Bonferroni correction. Moreover, we used SF analysis proposed by Mario Cortina-Borja et al.^22^ to examine potential SNP-SNP interactions and associations with MS. The values of *p* < 0.05 were considered statistically significant.

## Conclusion

In conclusion, our study supports the hypothesis the SNPs of the genes involved in oxidative and nitrosative stress may have an impact on individual risk of developing MS. We have found that the genetic variants in the *SOD2* (c.47 T > C; rs4880), *CAT *(c.-89 A > T; rs7943316), *GPX4* (c.660 T > A; rs713041)*,* and *NOS2* (c.1823 C > T; rs2297518) genes may modulate MS occurrence. Therefore, these polymorphisms may be considered as potential independent biomarkers of MS diagnosis. Moreover, this knowledge could significantly contribute to developing in the future novel strategies that would protect from disease development and progression. While this is the first time the direction has been well defined, more research is needed.

## Data Availability

The data that support the findings of this study are available on request from the corresponding author [Angela Dziedzic; angela.dziedzic@biol.uni.lodz.pl].
